# The Role of Genetic Polymorphisms in Chronic Pain Patients

**DOI:** 10.3390/ijms19061707

**Published:** 2018-06-08

**Authors:** Nebojsa Nick Knezevic, Tatiana Tverdohleb, Ivana Knezevic, Kenneth D. Candido

**Affiliations:** 1 Department of Anesthesiology, Advocate Illinois Masonic Medical Center, 836 W. Wellington Ave. Suite 4815, Chicago, IL 60657, USA; tverdohlebtatiana777@gmail.com (T.T.); drivanaknezevic@gmail.com (I.K.); kdcandido1@gmail.com (K.D.C.); 2 Department of Anesthesiology, College of Medicine, University of Illinois, Chicago, IL 60612, USA; 3 Department of Surgery, College of Medicine, University of Illinois, Chicago, IL 60612, USA

**Keywords:** genetic polymorphisms, chronic pain, low back pain, fibromyalgia, migraine, neuropathic pain, pain medications

## Abstract

It is estimated that the total annual financial cost for pain management in the U.S. exceeds 100 billion dollars. However, when indirect costs are included, such as functional disability and reduction in working hours, the cost can reach more than 300 billion dollars. In chronic pain patients, the role of pharmacogenetics is determined by genetic effects on various pain types, as well as the genetic effect on drug safety and efficacy. In this review article, we discuss genetic polymorphisms present in different types of chronic pain, such as fibromyalgia, low back pain, migraine, painful peripheral diabetic neuropathy and trigeminal neuralgia. Furthermore, we discuss the role of CYP450 enzymes involved in metabolism of drugs, which have been used for treatment of chronic pain (amitriptyline, duloxetine, opioids, etc.). We also discuss how pharmacogenetics can be applied towards improving drug efficacy, shortening the time required to achieve therapeutic outcomes, reducing risks of side effects, and reducing medical costs and reliance upon polypharmacy.

## 1. Introduction

Approximately 44 million Americans are suffering from moderate to severe chronic pain [1]. The cost of chronic pain management is estimated to reach 100 billion annually with private insurers paying the largest share of incremental costs (from 112 to 129 billion). Medicare’s cost is estimated to be from 66 to 76 billion with out-of-pocket health care expenditures, constituting an additional 44–71 billion [1,2]. Furthermore, the global prevalence for opioid users is estimated to be 32.4 million [2]. Each year, approximately 70,000–100,000 people die worldwide from opioid overdoses [3]. According to the U.S. Drug Enforcement Administration (DEA), “overdose deaths, particularly from prescription drugs and heroin, have reached epidemic levels” with almost half of all opioid overdose deaths in 2016, involving prescription opioids [4].

Treatment of the chronic pain patients has a high risk of having poor outcomes, especially when patients are treated with opioid medications. Individualized approaches to treating chronic non-cancer pain are paramount in improving patient’s quality of life and functionality, and in preventing severe adverse effects from opioid therapy (hyperalgesia, misuse, overdose and death).

In 2000, the completion of the Human Genome Project Research revolutionized the world of medicine; it opened up a new scope for disease process diagnosis, drug development and practicing medicine using an individualized approach. Advances in research, technology and policies that empower patients, also enable the development of personalized medicine in the field of pain management.

## 2. Genetic Polymorphisms Influence on Chronic Pain Conditions

When health care providers are diagnosing and treating chronic pain patients, a multitude of factors influence both processes, such as age, sex, ethnicity, comorbidities, multi-drug therapy and lifestyle. All of these factors, to a certain degree, including the genetic contribution to different types of pain and the genetic influence on drug efficacy and safety, will influence the outcome of pharmacotherapy.

### 2.1. Fibromyalgia

Fibromyalgia (FM) is a disease herald by chronic widespread pain, without any obvious organic lesions. It has an incidence of 2% in the general population and affects women predominantly [5]. A variety of factors are involved in the pathophysiology of FM, including psychological, genetic and environmental [5]. Studies have shown FM to have a 50% heritability; parents and siblings exhibit clinical evidence of FM with abnormal muscle consistency on palpation, and offspring of FM-diagnosed mothers are found to have a high incidence of FM [5]. Furthermore, FM patients demonstrate presence of familial aggregations of psychological features, such as depression and certain personality traits [6].

Arnold et al. performed linkage analysis with a genome-wide linkage scan for FM in a cohort of 116 families [7]. Coordinates of serotonin transporter gene (*SLC6A4*) and transient receptor potential vanilloid 2 genes (*TRPV2*) are coinciding with a signal in the chromosome 17p11.2–q11.2 regions, suggesting the linkage of these two genes with FM [7]. Furthermore, FM is associated with interference in serum and cerebrospinal fluid (CSF) serotonin metabolism and neurotransmission, demonstrating significantly lower levels of 5-hydroxytriptamine (5-HT) in serum and CSF [8]. Studies have also demonstrated an association between serotonin transporter (5-HTT) gene polymorphisms and severe symptoms of depression and anxiety-related traits in FM [9].

Catechol-*O*-methyltransferase (COMT), the enzyme involved in the degradation of dopamine, norepinephrine and epinephrine, has been found to contribute to FM susceptibility through genetic polymorphism [10]. One of the most studied short nucleotide polymorphisms (SNPs) of this enzyme is the *Val15Met* (*rs4680*). Studies have found associations between *rs4680* and FM susceptibilities in Brazilian, Spanish, Turkish, and Israeli populations, whereas a Korean population displayed COMT SNP *rs4818* and *rs4633* association with FM [8,11].

Other associated genes involved in the susceptibilities of FM are: Dopamine receptor D4 (*DRD4*), monoamine oxidase (*MAO-A*), β-2 adrenergic receptor, guanosine triphosphate cyclohydrolase (*GTPCH*) (involved in the dopamine, serotonin, and nitric oxide production), γ-aminobutyric acid (*GABA*), a receptor β3 subunit (*GABRB3*); sodium channel NaV1.7 (*SCN9A*); apolipoprotein (*APOE*); myelin transcription factor 1-like (*MYT1L*) (involved in neuronal differentiation) and neurexin (*NRXN3*), a synaptic scaffolding stabilizer in glutamate and GABA neurotransmission [12]. Additionally, Inanir et al. studied the angiotensin converting enzyme (*ACE*) and methylenetetrahydrofolate reductase (*MTHFR*) gene polymorphisms in 200 FM Turkish patients [13]. The authors concluded that ACE I/D polymorphisms are associated with an increased susceptibility for FM. In addition, it was determined that *MTHFR C677T* polymorphisms were associated with symptoms of stiffness and xerophthalmia [13].

FM is not a homogenous condition; although it has demonstrated a predominant association with catecholaminergic (COMT enzyme) and serotoninergic system (HTR2A) variations [14], there are more genetic variations to be thoroughly studied. The current drug therapy, either in combination or as monotherapy, appears to have limited efficacy and increased incidence of side effects in individuals carrying genetic polymorphisms (Table 1). Considering that FM is a multisystem disorder with a strong psychological association, cognitive behavioral therapy continues to play a significant role in pain management in these patients. Knowing the incidence of the main genetic polymorphisms in specific populations associated with FM, researchers should facilitate the development of novel targeted pharmacotherapy.

### 2.2. Chronic Low Back Pain

The genetic factors increase the susceptibility for chronic low back pain (CLBP) by 50%. The risk for persistent CLBP and sciatica is increased by the SNPs *A118G*, *rs1799971*, in the opioid receptor µ 1 (*OPRM1*) gene [15]. A study on 252 patients with lumbar disk herniation and sciatica, conducted by Olsen et al., demonstrated an interaction between gender and *OPRM1 A118G* genotype during recovery of low back pain and sciatica [16]. Women with the */G genotype report more pain and demonstrated slower recovery, compared to men with */G at 12 months after disk herniation [16].

A substitution of glutamine to tryptophan (Gln326 to Trp) in the α2 chain of collagen IX (Trp2 allele) showed association with inherited lumbar disk disease (LDD) [17]. The Trp2 allele is found in 4% of Finnish patients with LDD, and was not found in the control group. Linkage analysis demonstrates the presence of allele in a dominant fashion in the families of the carrier patients, which suggests that the Trp2 allele is present in dominantly inherited LDD [17]. The mechanism, by which the substitution of arginine to tryptophan (Arg103 to Trp) in the α3 (IX) chain increases the risk for LDD, can be explained by the fact that Trp is a hydrophobic amino acid, which is not characteristic for collagen IX—an essential structural component of the nucleus pulposus, annulus fibrosus and vertebral body endplate [18].

COMT polymorphisms was demonstrated to influence pain phenotypes in women with chronic pain, which reveals that gender is a significant factor in tailoring pain management [6]. COMT SNPs showed association with increased pain sensitivity in patients following surgery for low back pain [6]. A study by Jacobsen et al. reported an association of *rs4680* with long-term disability and delayed recovery in European patients with LDD, whereas another study showed that *COMT* SNPs *rs4680* has been associated with improvement of CLBP after treatment [19]. The highest disk degeneration grade was observed in patients with one minor allele (*CG* for *rs165656* and *CT* for *rs4633*), but none was observed in patients with two minor alleles (*CC* for *rs165656* and *TT* for *rs4633*) [20]. COMT polymorphism continues to represent a challenge in identifying the specific SNPs associated with predisposition and severity of LDD.

An increased risk for LDD has been associated with caspase-9 polymorphisms (CASP9)—an enzyme that activates the intrinsic pathway of apoptosis, with elevated levels of growth differentiation factor 5 (*GDF5*), with the variable nucleotide tandem repeat (VNTR) in the chondroitin sulfate-1 encoding domain of the aggrecan gene (*ACAN*), with matrix metalloproteinase SNPs [32]. It is significant that genetic polymorphisms play a tremendous role in identifying the patients at risk. Considering that LDD has a predominant genetic etiology, it is essential to apply personalized therapy in these patients. In the era of the present opioid epidemic, chronic pain management will involve DNA screening for genetic polymorphisms that will optimize personalized treatment in the near future (Table 1). The current drug therapy can be individualized for certain patients without the need for genetic testing; this can be accomplished through a “trial” period of medications with different metabolic pathways, different mechanisms of action or different dosages. The available drug therapy is focused on treating patients that already suffer from some types of chronic pain; however, the main focus should be on how to decrease the association of certain SNPs with increased susceptibility to these types of pain, increased pain sensitivity and increased opioid consumption, and this is where development of novel gene-targeted therapies is needed.

### 2.3. Migraine Headaches

Similar to the pain conditions mentioned above, migraine headache has a 50% genetic component to its etiology [21]. The increased susceptibility to migraine is supported by genetic polymorphisms, such as encoding endothelin type A receptor (*EDNRA*), encoding *MTHFR*, encoding endothelial nitric oxide synthase (*NOS3*), encoding *ACE*, encoding β-2 transforming growth factor (*TGFB2*), encoding β-2 transforming growth factor receptor (*TGFBR2*) and neurogenic locus notch homolog protein 3 (*NOTCH3*) (a receptor that is involved in vascular development and integrity) [12]. These gene variants are thought to be involved in the vascular etiology of migraine, which is explained by a deficient response to oxidative stress, and an imbalance between vasodilator and vasoconstrictor mediators, with subsequent elevation in pro-inflammatory cytokines [33]. The hormone-regulated etiology of migraine is supported by the estrogen receptor 1 (*ESR1*) genetic variability [22]. *ESR1* is a receptor and a nuclear transcription factor that regulates the endogenous steroid hormones, and promotes cell proliferation and differentiation, including neuronal cells. Therefore, SNPs in the *ESR1* gene can interfere with these functions. Individuals that displayed CC or CG genotypes, and CC combined phenotypes, have an approximately 50% increased risk for migraine in a Caucasian population [12]. In addition, *ESR1 rs1801132* (325C>G) is positively associated with migraine in a Spanish population. *ESR1 rs2228480* (594G>A) carries a higher risk for migraine in an Australian population, whereas *ESR1* rs2234693 is associated with migraine susceptibility in a Chinese population [23]. The neuronal etiology of migraine is described by increased ascending signaling and diminished descending inhibitory signals [24]. The glutaminergic, dopaminergic, serotoninergic, and GABA-ergic systems have been implicated in the neuronal etiology of migraine, with each of them presented with genetic variations of proteins, enzymes, receptors and ion channels [21]. High levels of plasma homocysteine are demonstrated to have a strong association with an increased risk for migraine [25]. Genetic variations of *5,10-MTHFR C677T* (*rs1801133*), *A1298C* (*rs1801131*), and nicotinamide-*N*-methyltransferase (*NNMT*), an enzyme that catalyzes the transfer of methyl group from S-adenosylhomocysteine to nicotinamide, are shown to significantly increase the plasma level of homocysteine [26].

The association between five COMT polymorphisms and migraine with or without aura and tension-type headache was studied in a Japanese sample population [27]. No association of the selected alleles (*rs4633*, *rs6267*, *rs4680*, *rs6270*, *rs740602*) was found, although the authors have concluded that an association between COMT genetic polymorphisms and migraine pathogenesis cannot be entirely excluded [27] (as shown in Table 1). The genome-wide association studies (GWASes) of migraine have identified many genes associated with migraine. The latest GWAS on a broadly defined headache phenotype has identified 28 loci associated with the phenotype. Among them, 14 loci have been previously reported associating with migraine, while 14 loci are newly identified [34].

### 2.4. Painful Diabetic Peripheral Neuropathy

Painful diabetic peripheral neuropathy (DPN) is a disabling complication in diabetic patients, which negatively affects their quality of life [35]. Two of the most accepted risk factors, contributing to the development of painful DPN, are a prolonged diabetic condition and a poor glycemic control. Unfortunately, the current available pharmacotherapy does not provide sufficient pain management for this condition, due to its multifactorial pathophysiology (e.g., environmental, metabolic, and genetic) [28]. Blesneac et al. conducted a study of 189 DPN patients screening for genetic variants of *Nav1.7* and its association with painful DPN. Ten patients with painful DPN were identified for 12 rare variants of *Nav1.7*; these patients were diagnosed for a significantly shorter period, reported more severe burning pain, and were more sensitive to deep pressure [28]. Furthermore, out of these 12 genetic variations, five variants were previously associated with idiopathic small fiber neuropathy and primary erythromelalgia. The authors concluded that rare variants of *Nav1.7* can increase the risk of developing painful DPN, and that further research is needed to understand how channel dysfunction contributes to the mechanism of specific pain phenotypes [28].

Meng et al. conducted a GWAS on 961 patients with peripheral DPN with 3260 diabetic patients as controls [29]. The authors found two loci that may be involved in DPN, *Chr1p35.1* (ZSCAN20-TLR12P) at *rs71647933* in females and *Chr8p23.1* at *rs6986153* in males. In addition, males displayed a higher heritability for DPN (30%), compared to females (14.7%) [29].

APOE is an important component in mitigating the cellular oxidative stress and inflammation response. Animal models with sciatic nerve injury demonstrate an increased level of APOE. Conversely, animal models with sciatic nerve injury and a decreased level of APOE, displayed impaired nerve regeneration [30]. On the other hand, human studies have been inconsistent and have failed to demonstrate an association of DPN development and APOE genetic polymorphism. The vascular endothelial growth (VEGF) factor is involved in the process of angiogenesis; studies have failed to demonstrate an association of its genetic polymorphism with vulnerability for DPN [30].

Despite addressing the modifiable risk factors for developing peripheral DPN (smoking cessation, controlling hypertension, weight loss, preventing and treating hypercholesterolemia), and supporting a tight glycemic control, studies have not demonstrated a decreased incidence of associated neuropathic pain in diabetic patients [31]. Genetic association represents an independent risk factor, and patients at risk should be recognized early and addressed clinically (Table 1).

### 2.5. Trigeminal Neuralgia

The serotonin transporter gene plays an important role in the regulation of serotonin transporter, which inhibits the release of serotonin into the synaptic cleft, terminating its neurotransmission [36]. Serotonin transporter gene is well studied in different psychiatric conditions and their therapeutic management. Recently, this regulator gained interest in chronic non-cancer pain conditions and analgesic response to drug therapy [36]. Cui et al. found a close association between *5-HTT*-linked polymorphic region (*5-HTTLPR*) and the susceptibility and severity of trigeminal neuralgia (TN) in 244 Chinese patients, and a close association with treatment response to carbamazepine monotherapy [36]. However, the small sample size and more profound studying of a molecular mechanism of *5-HTTLPR* were determined as study limitations (Table 1).

## 3. Genetic Polymorphisms and Gene Products Influence on Pain Medications

Metabolism of many medications involves Cytochrome P450 (*CYP450*), which are membrane-associated proteins in the endoplasmic reticulum [37]. Protein expression, structure and consequently protein function are altered by genetic polymorphisms. Variability in response to analgesic medications is dependent on genetic variations in: metabolic activation of pro-drugs, degradation of active components and transmembrane transport systems [38].

### 3.1. Duloxetine

Pharmacotherapy of FM involves diverse classes of medications, such as antidepressants, anticonvulsants, non-steroidal anti-inflammatory and narcotic medications [39]. The majority of these drugs are metabolized through *CYP450* enzyme subsets (*CYP1*, *CYP2*, and *CYP3*) [38]. Duloxetine is a serotonin and norepinephrine reuptake inhibitor and weak dopamine reuptake inhibitor approved by the Food and Drug Administration (FDA) for treating FM, neuropathic pain, depression, and generalized anxiety disorder [40]. *CYP2D6*, *CYP2C9*, and predominantly *CYP1A2*, contribute to duloxetine metabolism into its active forms: glucoronate conjugate 4-hydroxy and sulfate conjugate 5-hydroxy-6 methoxy duloxetine. Inhibition of some of these enzymes can lead to interactions and changes in the outcome of therapy for the FM patient population [41]. It is known that duloxetine is a moderate inhibitor of the *CYP2D6* enzyme subset. The administration of duloxetine to *CYP2D6* poor metabolizers (PMs), along with inhibition of *CYP1A2*, demonstrated clinically significant higher duloxetine exposure, emphasizing that co-administration with a potent *CYP1A2* inhibitor should be avoided. However, a *CYP2D6* PM in the absence of *CYP1A2* inhibition does not require dose adjustments, despite the 1–3 fold increase in plasma duloxetine [42]. Furthermore, co-administration of duloxetine with a *CYP2D6* substrate (e.g., risperidone, aripiprazole, metoprolol, etc.) is not recommended, due to potential increases in toxic levels of the substrates [38].

### 3.2. Amitriptyline

Amitriptyline, a tricyclic antidepressant (TCA) prescribed for FM, is metabolized into its active compound nortriptyline through isoenzyme *CYP2C19*, and into hydroxyl metabolites through *CYP2D6* [43]. Additionally, tramadol used for FM for short-term opioid therapy, is primarily metabolized through *CYP2D6* to pharmacologically active metabolite desmethyltramadol [44]. Genetic polymorphisms of *CYP2D6* result in reductions of desired therapeutic effects or increased incidence of side effects [36].

### 3.3. Nonsteroidal Anti-Inflammatory Drugs (NSAIDs)

Pharmacotherapy of CLBP includes classes of medications that extensively depend on *CYP450* metabolism. *CYP2C9* enzyme contributes to the oxidation and metabolism of NSAIDs, and *N*-demethylation of some antidepressants (e.g., amitriptyline and fluoxetine) [41]. There are several reports stating that the carriers of CYP2C9*3 genetic polymorphisms have an increased risk for gastrointestinal bleeding [45]. Generally, the efficacy of NSAIDs is least likely to be measured by pharmacogenomics studies, and more emphasis is placed on the adverse effects in relation to *CYP2C9* genetic variations [38].

### 3.4. Opioids

In contrast to *CYP2C9*, the genetic polymorphisms of *CYP2D6* have been of substantial interest in chronic pain management, particularly due to a bimodal distribution of individuals classified as PMs and ultra-rapid metabolizers (UMs) [37]. This distribution varies in certain ethnicities, with some predominantly displaying a PM phenotype (6% of a Caucasian population), and others having a higher incidence of an UM phenotype (29% of Ethiopians and 21% of a Saudi Arabian population) [46].

Hydrocodone is a semi-synthetic opioid medication that depends on *CYP2D6* enzyme demethylatilation into hydromorphone (stronger µ receptor binding), and on the *CYP3A4* enzyme for metabolism into norhydrocodone [47]. Although data is inconsistent with respect to hydrocodone efficacy and safety in PM phenotypes, it is strongly recommended to avoid co-administration of a *CYP3A4* inhibitor in these patients, due to an increased risk of a drug–drug interaction [48].

*CYP3A4* enzyme metabolizes oxycodone by N-demethylation to noroxycodone, and the *CYP2D6* enzyme catalyzes *O*-demethylation of oxycodone to oxymorphone, which is 14-fold more potent than the parent oxycodone [49]. *CYP2D6* genetic polymorphisms have a major effect on analgesic efficacy and the side effect profile of oxycodone; *CYP2D6* UM displays an increased analgesic effect and toxicity, whereas *CYP2D6* PM demonstrates reduced oxycodone efficacy [49]. Furthermore, a drug–drug interaction (DDI) is of major concern, where *CYP3A4* inhibitors can significantly increase the analgesic effect and toxicity of oxycodone in patients with *CYP2D6* UM phenotype [38].

SNP 118A>G (*rs1799971*) on chromosome 6 in the *OPRM1* gene coding for µ-opioid receptor leads to a substitution of aspartate for asparagine, altering the N-glycosylation of the receptor protein [50]. This alteration can influence the patients’ response to opioid analgesia. The Asian population demonstrated a higher frequency of SNP 118A>G, compared to Caucasian patients, which was concluded to be associated with increased opioid dose requirements for post-operative pain in the former individuals. In a cohort of 1000 women following breast cancer surgery, the 118A>G variation demonstrated a strong association with the amount of oxycodone needed for achieving adequate analgesia [50]. Although no association was found between 118A>G polymorphisms and total oxycodone consumption in the first 20 h post-surgery, the increased amount of oxycodone needed in the first stage of analgesia most likely reflects the amount of oxycodone needed to reach the central µ-opioid receptors. Some studies have demonstrated an association between 118A>G polymorphisms with increased opioid consumption, whereas a meta-analysis has disputed this association, concluding that 118A>G is of minor importance [50]. Nonetheless, a strong association between *OPRM1 rs589046*, and *OPRM1 rs563649* with poor oxycodone analgesic response has been demonstrated [46]. Other SNPs associated with poor oxycodone responses were found at the G allele of *OPRD1 rs419335* that showed decreased analgesia, following oxycodone for visceral heat stimulation. The T allele of *OPRM1 rs563649* carriers and C allele of *OPRM1 rs589046* demonstrate decreased oxycodone efficacy to experimental skin heat stimulation.

### 3.5. Prophylactic Drugs Treatment for Migraine

Prophylactic treatment of migraine is non-specific for the condition, and a “trial and error” approach is more typically adopted. *Rs2274316* is nominally associated with a lower effects of beta-blocker use in migraine only (MO) patients, whereas *rs2651899* is nominally associated with increased effects in conditions of migraine with typical aura (MTA) [51]. Furthermore, angiotensin II receptor-antagonists are found to display a lower effect for MTA and MO patients with rs11172113, whereas alleles rs6790925 displays a nominal association with a higher effect in patients with “all migraine” and in MO [52]. Finally, anti-epileptic prophylactic medication has a lower effect in MO patients with *rs2651899* allele, whereas *rs10504861* is nominally associated with a higher effect in “all migraine” patients [53]. Migraineurs would benefit significantly from individualized prophylactic therapy, since every drug must go through three months of “trial and error” before demonstrating its efficacy. Furthermore, migraine etiology is influenced by environmental stimuli, such as temperature, light and mechanical stimuli, all of which contribute to central sensitization of the migraine pain cycle through vanilloid receptors. Developing new medications targeting at these receptors could possibly alleviate the suffering of this population [12].

## 4. Clinical Applications of Pharmacogenetics

### 4.1. Pain Perception Assessment

Although genetic testing of COMT haplotypes has been proven to predict the level of pain perception, it is not a frequently used clinical practice tool. However, genetic testing for pain perception would potentially guide physicians’ clinical decision making for a better outcome in patients suffering from various chronic pain conditions, either for initiating a treatment or for re-evaluating an ongoing treatment.

Sharma et al. conducted an observational study on the impact of genetic testing on pain perception in the setting of chronic non-cancer pain management [54]. COMT SNPs *rs6269*, *rs4633*, *rs4818* and *rs4680* were genotyped, followed by pain perception haplotypes and score calculation: low pain perception (G_C_G_G)-1–2, moderate pain perception (A_T_C_A)-3, and high pain perception (A_C_C_G)-4–5 [55]. The data for 134 patients was consistent with the genotypic pain perception and the self-reported pain score in 81–90% of the cases. There were 92 treatment decisions made based upon genotypic pain perception, which consisted of decreasing, discontinuing, maintaining the medication dose, as well as decisions for further diagnostic testing (Magnetic Resonance Imaging (MRI), X-ray), and decisions for pursuing other interventions and therapies (physical therapy, cognitive behavioral therapy, interventional pain management, specialty consultations and research studies). The outcome of these actions was measured as patient’s clinical status change (improved, unchanged, and worsened) with 72% improvement based on pharmacologic interventions, whereas 69% of patients demonstrated clinical improvement in response to non-pharmacologic interventions [54]. Although the study has some important limitations (small sample size, mixed population sample and no control group), it provides an important outlook for personalized treatment as a routine clinical practice with improved decision-making and better outcomes. 

### 4.2. CYP450 2D6 Genotyping

Knowing the patient’s *CYP2D6* phenotype can help guide physicians in selecting the most appropriate medication and dosage for increased efficacy and decreased side effects [56]. The Dutch Pharmacogenetics Working Group (DPWG), established in 2005, has a primary goal of developing pharmacogenetic-based therapy for physicians and pharmacists in different areas, and to assist drug prescribers and pharmacists with computerized system recommendations for drug prescription and automated medication surveillance [57]. Recommendations are developed based on peer-reviewed literature published in the last two decades, for which two parameters are clearly defined: the level of evidence of the gene-drug interaction and the clinical significance of the potential adverse drug event, decreased therapeutic response, or other clinical effects resulting from gene–drug interactions. A score system is generated based on the level of evidence for gene–drug interactions (a score of four being the highest level of evidence). Furthermore, clinical relevance for the above-mentioned parameters is also graded from AA (lowest clinical impact) to F (the highest clinical impact) [57].

Clinical Pharmacogenetics Implementation Consortium (CPIC), along with DPWG, established recommendations for pharmacogenetic-based dosing for patients with common *CYP2D6* phenotypes [58]. The clinical utility of drug–gene correlations was assessed for patients on antipsychotic or antidepressant medications, such as selective serotonin reuptake inhibitors (SSRIs), non-selective reuptake inhibitors (NSRIs), and TCA [59]. For the *CYP2D6* PM phenotype, the recommendations were to reduce the dose for clomipramine, haloperidol, (both 50% reduction), doxepin, nortriptyline (both 60% reduction) and imipramine (70% reduction) due to the high risk of toxicity; whereas for amitriptyline, risperidone, and venlafaxine, insufficient evidence was reported for making dose adjustment [60]. Commonly, some of these drugs are prescribed in different chronic pain conditions, such as FM and chronic neuropathic pain, and for a great majority of chronic pain patients, these medications are used for treating concomitant psychiatric conditions (e.g., depression and anxiety). For *CYP2D6* intermediate-metabolizer (IM) patients, dose reductions for doxepin, amitriptyline, imipramine and nortriptyline range between 20–50% [61]. In *CYP2D6* UM, the range of dose adjustments for doxepin, imipramine, nortriptyline, venlafaxine and tramadol widely varies from 30–150%, attributed to significantly greater gene copies [62]. With regards to pain management, both CPIC and DPWG have published pharmacogenetic-based dosing guidelines. Oxycodone, as one of the *CYP2D6* metabolic pathway drugs, was reported to have insufficient evidence for dosing reduction in patients with *CYP2D6* PM and IM phenotypes, whereas tramadol use in *CYP2D6* UM has a wide range of dose adjustments [63]. The DPWG has determined that *CYP2D6* PM treated with codeine receives a score of 4B for clinical impact, whereas patients with *CYP2D6* UM phenotype are attributed a score of 3F for clinical impact [64]. The recommendations to choose an alternative medication in such patients should be considered, with a different or non-*CYP2D6* predominant metabolic pathway. 

Hocum et al. conducted another notable study that demonstrated a potentially considerable clinical implication of *CYP* genetic polymorphisms in a mixed-race study population [37]. They have evaluated the polymorphisms of the most common *CYP* isoenzymes (*CYP2D6*, *CYP2C9*, *CYP2C19*, *CYP3A4*, and *CYP3A5*), and then categorize subjects into the four phenotypes: UM, extensive metabolizer (EM), IM and PM. The phenotypes, along with the submitted individuals’ medications list, was analyzed by web-based software and categorized into three types of interactions: DDIs, drug–gene interactions (DGIs), and drug–drug–gene interactions (DDGIs) [37]. Among the 22,885 individuals who underwent pharmacogenetic testing, 93% had two or more risk-associated phenotypes, with increased prevalence for IM and PM phenotypes. Furthermore, in 16,924 subjects with reported severe interactions, the recommendations were to “change” or “consider changing” the medication regimen; only 47% of these subjects had a genetic component to the cause of interactions, out of which 24.6% of all “change” or “consider” interactions were DGIs, and 22.4% were DDGIs [37]. Even more interesting, the most frequent and severe interactions were found in the older population, compared to the younger subjects, and with the former taking 10.5 medications on average, compared to the latter with an average 7.2 medications per patient. Considering that a significant number of patients with high-risk phenotypes, and increased prevalence of DGIs and DDGIs, the authors concluded that patients, who are taking a large number of medications despite their age, would benefit most from clinical pharmacogenetic testing [65].

### 4.3. Methadone Initiation Assessment

Approximately one-third of opioid-related prescription drug deaths are attributed to methadone [66]. Methadone is a well-established treatment for opioid addiction; however, it requires exceptional caution when initiating therapy. The major contributory risks for serious adverse events are the overestimation of tolerance and lack of profound pharmacokinetic evidence for plasma drug concentration for successful treatment. Therefore, accumulation to toxic levels and poor metabolism can lead to death in the first few weeks of initiating methadone therapy. Genetic variations are believed to play an important role in the wide interindividual pharmacokinetic and pharmacodynamic response to methadone. Methadone undergoes *CYP* enzyme system metabolism, primarily by *CYP2B6* enzyme subset, which has demonstrated high polymorphisms [67]. Testing for *CYP2B6* SNPs before initiating methadone therapy may identify patients at risk for poor treatment responsiveness, as well as patients at high risk of drug toxicity from poor metabolism [68].

### 4.4. Opioid Addiction Genotyping

A pharmacogenetic study conducted by Christoffersen et.al examined whether certain genotypes are associated with sudden death due to opioid addiction. They found no statistically significant differences in the frequency of the TT genotype of *rs1045642* in *ABCB1* between the deceased opioid addiction (DOA) population of patients, living patients with active opioid addiction (LOA) getting opioid replacement therapy and healthy volunteers [69]. However, a statistically lower frequency of AG and TT in the ATP-binding cassette sub-family B member 1 (*ABCB1*) *rs9282564* in DOA compared to LOA was found when adjusted for age and sex, and lower *COMT rs4680* AA genotype frequency in DOA (25%), compared to LOA (35%), and healthy volunteers (31%), suggesting lower risk of death in patients with opioid addiction [69]. Further research is required before establishing a causal relationship between these genetic variants, and if findings can be replicated, the personalized treatment through pharmacogenetic testing can help find high-risk individuals, prevent sudden death and help resolve the consequences of the opioid epidemic.

The impacts of different SNPs in clinical outcomes are listed in Table 2, which is summarizing the main features of studies that analyze the role of different SNPs in chronic pain.

## 5. Conclusions

Genetic polymorphisms have a significant clinical impact in diagnosing and treating different types of chronic pain; it can manifest as increased pain sensitivity in certain individuals, increased opioid consumption, poor therapy response, high incidence of side effects and disease process complications.

Health care professionals have a significant number of tools available to use to manage patients’ chronic non-cancer pain using an individualized approach. Physicians have a low threshold for suspecting decreased efficacy or increased toxicity attributed to age, concomitant advanced or poorly managed disease processes, drug–drug interactions, inappropriate dosing of medication and even metabolic phenotypes. After eliminating and correcting these factors without significant improvement, physicians often do not proceed to use advanced available diagnostic tools, mainly due to their cost and lack of clear guidelines.

## Figures and Tables

**Table 1:** Pharmacogenomics of disease processes and incidence of the main genetic polymorphisms in specific populations.

Chronic Pain Type	References	Receptors and Mediator Channels Involved in Disease Susceptibility	Pharmacogenomics of Pain Medications	Possible Clinical Implications	Associated Population
**Fibromyalgia**	[8,9,10,13,14]	- *COMT-Val15Met* (*rs4680*) - *COMT-Vall5Met* (*rs4818*) and (*rs4633*) - *DRD4* - *MAO-A* - β-2 adrenergic receptor - *GTPCH* (*GABA*) A receptor, - *GABRB3* - Sodium channel NaV1.7 (*SCN9A*) - *APOE* - *MYT1L* - *NRXN3* - *MTHFR C677T*	- Duloxetine metabolism through *CYP2D6, CYP2C9*, and predominantly *CYP1A2* - Amitriptyline metabolism into nortriptyline (through *CYP2C19*), and hydroxyl (through *CYP2D6*)	- Avoid co-administration of duloxetine with a *CYP2D6* substrate (e.g., risperidone, aripiprazole, metoprolol, etc.) due to potential increases in toxic levels of the substrates	- Brazilian, Spanish, Turkish, and Israeli *Val15Met* (*rs4680*) - Korean (*COMT*–SNP *rs4818* and *rs4633*)
**Chronic Low Back Pain**	[15,16,17,18,19,20]	- *OPMR1*-SNPs *A118G* (*rs1799971*) - Gln326 to Trp - Arg103 to Trp - *COMT* SNPs *rs4680* - *COMT-CG* for *rs165656* and *CT* for *rs4633* - *CASP9* - Elevated levels of *GDF5* - VNTR in the chondroitin sulfate-1 encoding domain of the *ACAN* - Matrix metalloproteinase SNPs	- NSAIDs metabolism through *CYP2C9* (increased risk for adverse effects) - Opioids metabolism through *CYP2D6* and *CYP3A4* (an increased risk for adverse effects in UM, and decreased analgesic efficacy in PM) - Poor Oxycodone analgesic in *OPRM1 rs589046*, and *OPRM1 rs563649*	- Pain perception assessment - *CYP2D6* genotyping based on scores for level of evidence and clinical relevance - Genotyping for Methadone treatment initiation - Genotyping for at risk for addiction population	- Finish (Gln326 to Trp)
**Migraine**	[21,22,23,24,25,26,27]	- *EDNRA* - encoding *MTHFR* - encoding *NOS3* - encoding *ACE* - encoding *TGFB2* - encoding *TGFBR2* - *NOTCH3* - *ESR1 rs1801132* (325C>G) - *ESR1 rs2228480* (594G>A) - *ESR1 rs2234693* - *5,10-MTHFR C677T* (*rs1801133*), *A1298C* (*rs1801131*) - *NNMT*	- *Rs2274316*-nominally lower effect of β-blocker in migraine only (MO) - Angiotensin II receptor-antagonists—lower effect for migraine typical aura (MTA) ± migraine without aura (MO) patients with *rs11172113*	- Individualized prophylactic treatment	- Spanish (*ESR1 rs1801132* (325C>G) - Australian (*ESR1 rs2228480* (594G>A) - Chinese (*ESR1 rs2234693*)
**Diabetic Painful Peripheral Neuropathy**	[28,29,30]	Genetic variants of *Nav1.7*	- Duloxetine metabolism through *CYP2D6*, *CYP2C9*, and predominantly CYP1A2 - Amitriptyline metabolism through nortriptyline (*CYP2C19*), and hydroxyl (*CYP2D6*)	- Correction of modifiable risk factors - Management of hyperglycemia - Avoid co-administration of duloxetine with a *CYP2D6* substrate (e.g., risperidone, aripiprazole, metoprolol, etc.) due to potential increase to toxic levels of the substrates	- Males (*Chr8p23.1* at *rs6986153*) - Females (*rs71647933*) - DPN-males—30% more compared to Females
**Trigeminal Neuralgia**	[31]	Serotonin transporter gene (*5-HTT*)-linked polymorphic Region (*5-HTTLPR*)	Association between poor carbamazepine response and *5-HTTLPR* polymorphisms	- Reassess poor carbamazepine therapeutic response	- Chinese (*5-HTTLPR*)

**Table 2:** Summarizing the main features of the studies that analyzed the role of different SNPs in chronic pain and their impacts in clinical outcomes.

Condition	Reference	Number of the Evaluated SNPs and Pathway	Aim	Significant SNPs
**Fibromyalgia**	[8]	*TRPV2* gene 3 positions [*rs3813768* (C>G), *rs8121* (C>T), *rs1129235* (C>A)] *TRPV3* gene 2 positions [*rs7216486* (G>A) and *rs395357* (C>T)]	Polymorphisms of the *TRPV2* and *TRPV3* genes associated with fibromyalgia in a Korean population.	*Rs395357* associated with symptom severity
**Fibromyalgia**	[10]	4 SNPs: *rs6269*, *rs4633*, *rs4818* and *rs4680* or *Val158Met* identified haplotypes designated as low (LPS), average (APS) and high pain sensitivity (HPS)	Pain sensitivity in fibromyalgia is associated with catechol-*O*-methyltransferase (*COMT*) gene	Met/Met genotype (*Val158Met* SNP)-increased pain sensitivity
**Low Back Pain**	[15]	*A118G* on the *OPRM1* gene	Association of mu-opioid receptor gene polymorphisms (*A118G*) with variations in morphine consumption for analgesia after total knee arthroplasty.	*G118* (*GG*) associated with significant morphine consumption
**Low Back Pain**	[19]	COMT *Val158Met* SNP	The COMT *rs4680 Met* allele contributes to long-lasting low back pain, sciatica and disability after lumbar disc herniation.	*Met/Met* genotype (*Val158Met*) was associated with significantly more pain compared to *Val/Met* and *Val/Val*
**Pain perception assessment**	[52]	*rs6269*, *rs4633*, *rs4818*, *rs4680*	COMT gene haplotypes are closely associated with postoperative fentanyl dose in patients.	COMT gene haplotype *ACCG* associated with significant fentanyl consumption
**Methadone initiative assessment**	[65]	CYP2B6*1/*1 (*n* = 21), CYP2B6*1/*6 (*n* = 20), CYP2B6*6/*6 (*n* = 17), CYP2B6*1/*4 (*n* = 1), CYP2B6*4/*6 (*n* = 3), CYP2B6*5/*5 (*n* = 2)	Methadone Pharmacogenetics: *CYP2B6* Polymorphisms determine plasma concentrations, clearance and metabolism.	CYP2B6*6-decreased metabolism and clearance in African-Americans

## Abbreviations

DEA	Drug Enforcement Administration
FM	fibromyalgia
SLC6A4	serotonin transporter gene
TRPV2	transient receptor potential vanilloid 2 genes
CSF	cerebrospinal fluid
(5-HT)	5-hydroxytriptamine
(5-HTT)	serotonin transporter
COMT	Catechol-*O*-methyltransferase
SNP	short nucleotide polymorphisms
DRD4	dopamine receptor D4
MAO	A-monoamine oxidase
GTPCH	guanosine triphosphate cyclohydrolase
GABRB3	GABA receptor beta 3 subunit
APOE	apolipoprotein MYT1L myelin transcription factor 1
CLBP	chronic low back pain
OPRM1	opioid receptor µ 1
LDD	lumbar disk disease
GDF5	growth differentiation factor 5
COMT	catecholaminergic enzyme
VNTR	variable nucleotide tandem repeat
NSAIDs	(nonsteroidal anti-inflammatory drugs)
ACAN	chondroitin sulfate-1 encoding domain of the aggrecan gene
EDNRA	encoding endothelin type A receptor
MTHFR	encoding methylenetetrahydrofolate reductase
NOS3	encoding endothelial nitric oxide synthase
ACE	encoding angiotensin-1 converting enzyme
TGFB2	encoding β-2 transforming growth factor
TGFBR2	encoding β-2 transforming growth factor receptor
VEGF	vascular endothelial growth factor
NOTCH3	neurogenic locus notch homolog protein 3
ESR1	estrogen receptor 1
MTHFR	methylenetetrahydrofolate reductase
DPN	painful diabetic peripheral neuropathy
5-HTTLPR	5-hydroxytriptamine transporter-linked polymorphic region
TN	trigeminal neuralgia
CYP450	cytochrome P450
TCA	tricyclic antidepressant
DPWG	Dutch Pharmacogenetics Working Group
CPIC	Clinical Pharmacogenetics Implementation Consortium
SSRIs	selective serotonin reuptake inhibitors
NSRIs	non-selective reuptake inhibitors
IM	intermediate-metabolizer
EM	extensive metabolizer
DDIs	drug-drug interactions
DGIs	drug-gene interactions
DDGIs	drug-drug-gene interactions
DOA	deceased patients with opioid addiction
LOA	living patients with opioid addiction
ABCB1	ATP-binding cassette sub-family B member 1
